# Preparation of High-Performance Metal-Free UV/Near Infrared-Shielding Films for Human Skin Protection

**DOI:** 10.3390/nano11081954

**Published:** 2021-07-29

**Authors:** Chih-Hao Liang, Ying-Jung Chen

**Affiliations:** 1 R&D Division, Walsin Technology Corporation, Kaohsiung 806, Taiwan; liangchihhao1983@gmail.com; 2 Department of Fragrance and Cosmetic Science, Kaohsiung Medical University, Kaohsiung 807, Taiwan; 3 Drug Development and Value Creation Research Center, Kaohsiung Medical University, Kaohsiung 807, Taiwan; 4 Department of Medical Research, Kaohsiung Medical University Hospital, Kaohsiung 807, Taiwan

**Keywords:** heat insulation, UV absorption, TiO_2_/SiO_2_ multilayers, ITO, fibroblasts, human skin

## Abstract

A series of metal-free UV/near infrared (NIR)-shielding coatings are successfully fabricated by shielded cathodic arc plasma evaporation (CAPE) and substrate-biased RF magnetron sputtering processes. The UV/NIR-shielding coatings comprising quarter-wave stacks of TiO_2_/SiO_2_ multilayers and high-conductivity sputter-deposited ITO films with a thickness in the range of 200–600 nm could block IRA and IRB radiations, respectively. The total thicknesses of UV/near infrared-shielding films are in the range from 375 nm to 1513.8 nm. The anatase-phase TiO_2_ films with absorption edge located at ~375 nm were deposited by shielded CAPE at ~100 °C. Further, the well-crystallized ITO films were found to have high free-electron concentrations (1.12 × 10^21^ cm^−3^), resulting in strong absorption of IRB due to the plasmon resonance absorption. The optimal optical design and ITO film thickness were investigated, and the TiO_2_(SiO_2_/TiO_2_)_3_ multilayer combined with an ITO film thickness of 400 nm was found to provide a high NIR-shielding rate of 94.8%, UVB to UVA-shielding rate of 92.7%, and average visible light transmittance of 68.1%. Further, human skin cells protected by a UV/NIR-shielding coating showed significantly decreased reactive oxygen species generation and inflammatory cytokine expression as compared to those of unprotected cells. The results demonstrate that the development of multifunction coatings have potential for transparent heat insulation windows and human skin protection against UV/IR radiations.

## 1. Introduction

Solar radiation near the Earth’s surface consists of a wide range of wavelengths, divided into three main ranges: UV, visible, and infrared (IR) radiations. While the IR light comprises ~54% of the total amount of the solar energy, the UV light (including the three types: UVA, UVB, and UVC) accounts for only 7% [1]. UVA (320–400 nm) can penetrate the epidermis layer to the dermis layer, leading to a photosensitive reaction via free radical generation [2]. Therefore, sunscreen materials are widely used in UVA (wavelength: 320–400 nm) and UVB (wavelength: 280–320 nm) protection for reducing the risk of skin cancers such as basal-cell carcinoma [3] and squamous-cell carcinoma [4]. Further, the conversion of the absorbed IR light into thermal causes an increase in the temperature of the window glass panes to a level as high as 50 °C, resulting in heat radiation into indoor spaces [5]. Thus, a large amount of energy is consumed in ensuring thermal comfort inside buildings using devices such as air-conditioning systems. Moreover, recent studies have indicated that IR light, which is subdivided into IRA (wavelength: 760–1400 nm), IRB (wavelength: 1400–3000 nm), and IRC (3000 nm to 1 mm) ranges, can also cause skin damage and aging under prolonged sunlight exposure [6]. IRA radiation comprises a major portion of the IR wavelengths of the sunlight, and has a 65% skin-penetration rate, greater than those of the IRB, IRC, and UV wavelengths [7]. Therefore, there is a high demand for development of multifunction coatings, which are combined with saving the energy and human skin protection against both UV and NIR irradiations.

To protect against the adverse effects of UV light, titanium dioxide (TiO_2_) powders are most widely used as sunscreen materials [8], because the band-to-band absorption edge of TiO_2_ in the anatase and rutile phases is located at ~388 nm and ~413 nm, respectively (i.e., the band gap is ~3.2 and ~3.0 eV for the anatase and rutile phases of TiO_2_, respectively) [9]. Moreover, TiO_2_-based materials have also attracted significant attention for many other possible applications (e.g., as photocatalysts for the degradation of pollutants, self-cleaning and/or superhydrophilic surfaces [10], solar cell [11], and optical coatings [12]). The properties required for TiO_2_-based optical coatings are highly transparent and have a high refractive index in the visible light range [12]. Previous studies have extensively investigated the effects of the TiO_2_ microstructure on their photocatalytic activities and optical properties. These studies indicated that amorphous TiO_2_ has negligible photocatalytic activity, which was attributed to the recombination of the photoexcited holes and electrons at the trap sites [13]. In addition, amorphous TiO_2_ films have a lower bandgap (~2.4 eV), resulting in visible light absorption [14]. In general, a high growth temperature (~400 °C) is required for the transition of the amorphous TiO_2_ phase to the crystalline anatase phase [15]. In the case of TiO_2_ films deposited by the direct current (DC) sputter deposition process at room temperature, a high-temperature thermal treatment (>300 °C) is usually required to obtain well-crystallized anatase-phase films with a high reflective index and high visible light transmittance [16]. Previously, an ion-assisted deposition process was used to increase the refractive index of TiO_2_ films; it was achieved by the enhanced reactivity and high energy of the Ti ions impinging on the surface of TiO_2_ film during the deposition [12]. Among these ion-assisted deposition processes, cathodic arc plasma evaporation (CAPE) is the most promising method for growing highly crystalline films at a low substrate temperature, which is due to the high degree of ionization and suitable ion kinetic energies in the range of 10–100 eV [12].

NIR-shielding coatings are designed to selectively reflect or absorb light in the NIR range and highly transmit in visible light range. For instance, thin metal coatings (such as Ag and Au thin film) have been applied on glass sheets for shielding the IR wavelengths, because they have high free electron densities, and hence have a the plasmon resonance absorption in the NIR region of ~1000 nm [17]. Although Ag coatings have excellent NIR-shielding properties, they are chemically unstable and less durable in high-humidity environments. To improve the stability of Ag layers in high-humidity environments, metal oxide/metal/metal oxide (such as MO_x_/Ag/MO_x_) multilayer coatings have been extensively explored as transparent energy-saving coatings [17,18]. However, Ag oxidation is a critical issue during the direct deposition of the oxide protection layer in an O_2_ reactive atmosphere on the Ag layer [19]. The Ag oxidation introduces large intrinsic stresses, leading to partial delamination of the film [20]; further, the visible light transmittance is also decreased due to considerable light absorption by the formed Ag oxides. The durability of the MO_x_/Ag/MO_x_ multilayer coatings in humid environments depends on the internal stress of the MO_x_ layers, and a higher internal stress renders the coatings less durable [21]. Moreover, studies on the thermal stability and oxidation resistance of MO_x_/Ag/MO_x_ multilayer structures have indicated that oxygen out-diffuses into the Ag layer and oxidizes the thin Ag layer, leading to the formation of cracks in the multilayer structures [22]. In contrast, transparent conductive oxides (TCO) films are promising candidates for developing IR-shielding coatings, because they are chemically/thermally more stable than metal layers. Recently, TCO films and nanoparticles have been developed to serve as IR-shielding materials. The IR-reflecting edge (plasmon frequency) usually depends on the free electron density, because the IR absorption is due to the plasmon resonance. Therefore, intensive efforts have been devoted to improve the plasmon resonance absorption by increasing the free electron densities in these materials. The TCO materials have been tailored by adjusting their dopant concentration, as demonstrated for Zinc-oxide-based materials doped with group IIIA elements (Al, Ga) [23,24,25], PMMA-ITO composite coatings [26], and indium tin oxide (ITO) films [27]. However, the low IRA-shielding rate of these films remains an issue, because the plasmon resonance absorption of the TCOs lies in the NIR region of >1400 nm.

The aim of this work is to improve the chemical/thermal stability of UV/NIR-shielding coatings. We developed a viable thin film deposition method for preparing metal-free transparent UV/NIR-shielding coatings. A multilayer structure of crystalline anatase-phase TiO_2_ and amorphous SiO_2_ is designed to reflect the IRA wavelengths, because these two materials are well-suited for this purpose owing to the large difference in their refractive indices and high visible light transparency. These oxide-based UV/NIR-shielding coatings consists of TiO_2_ and SiO_2_ quarter-wave stack multilayers. The high UV-light-absorption TiO_2_ films were grown at a low temperature using the shielded CAPE technique. Further, ITO films with a high free carrier density, which exhibit high IRB-shielding performance, were deposited by substrate-based radio frequency (RF) magnetron sputtering. These deposition techniques offer a low growth temperature to prevent the inter-diffusion between the multilayers. Thus, the novelty of this work is further improving chemical and thermal stability of UV/NIR-shielding coatings for human skin protection.

## 2. Materials and Methods

### 2.1. Fabrication of the Samples

IR-reflecting multilayer coatings of high-refractive-index TiO_2_ films and low-refractive-index amorphous SiO_2_ films were deposited on glass substrates using steered CAPE with a shielded net and sputtering deposition, respectively. An arc cathode for the Ti source and an RF sputtering system for the Si source were installed on each side of the chamber wall. A rotatable substrate holder was located midway between the two sources. The distances from the arc cathode and sputter source to the substrate holder were 350 and 100 mm, respectively. A circular Ti target (diameter: 4 inch) was fixed on the water-cooled circular arc source to improve thermal conduction. The steered arc process was used to prevent overheating, and thus decrease the emission of Ti microdroplets. Circular permanent magnets placed behind the circular cathode created a cycloidal path of the spot on the surface of the target. The magnetic field strength at the center of the cathode was 25 G, and it decreased gradually to 7 G at the edge of the cathode. An electrically grounded shielded net (SUS304-made, 30 mesh) serving as the anode was located at a distance of 200 mm from the cathode surface between the cathode and substrate to prevent the Ti microdroplets from reaching the substrate. TiO_2_ films were deposited in an O_2_ gas atmosphere at a deposition pressure of 2.5 Pa and arc current of 80 A. Before deposition, the chamber was evacuated to <4.0 × 10^−4^ Pa. SiO_2_ films were grown by RF magnetron sputtering using a circular Si target (diameter: 3 inch) in an Ar-O_2_ mixed gas atmosphere (O_2_/Ar = 0.4) at a sputtering pressure of 0.53 Pa and RF sputtering power of 250 W. Typical deposition rates were 1.2 and 1.3 nm/s for the TiO_2_ and SiO_2_ films, respectively. The substrate temperature was maintained at ~100 °C during the deposition and was monitored using a thermocouple. The thermocouple was fixed on the substrate hold to prevent the shift thermocouple position during loading/unloading samples and the distance between the tip of the thermocouple and the substrate was around 3 mm. ITO films were subsequently deposited on the glass substrates on the opposite side of the TiO_2_/SiO_2_ multilayers by RF magnetron sputtering using a circular target (diameter: 3 inch) consisting of 90 wt% In_2_O_3_ and 10 wt% SnO_2_. The films were deposited in an Ar atmosphere at the sputtering pressure of 0.53 Pa. An RF sputtering power of 150 W was used to generate the plasma. A bias voltage of 40 V was applied to the substrate using the RF power source. According to our previous studies, a negative bias of 40 V is a suitable condition for the ITO film growth, as it provides sufficient kinetic energy to enable the adatoms to reach the equilibrium position and for the relaxation of the residual stress. The substrate temperature was maintained at ~150 °C during the sputtering process. ITO films with thicknesses of 200–600 nm were deposited at the rate of ~1.5 nm/s.

### 2.2. Characterization of the Samples

Film thicknesses were measured using a stylus surface profilometer (Tencor). An n&k analyzer (model 1200, n&k Technology, Santa Clara, CA, USA) was used to measure the refractive index (*n*) of the TiO_2_ and amorphous SiO_2_ films in the wavelength range of 190–1000 nm. The *n* values were found to be 2.25 and 1.45, respectively, for the TiO_2_ and SiO_2_ films. The optical transmission measurements were obtained using a UV-visible near-infrared spectrophotometer (200–2500 nm) (LAMBA 750, Perkin-Elmer, Inc., Spokane, WA, USA) in a double-beam configuration. The surface morphology of the coatings was observed using a scanning electron microscope (SEM, JSM-6330TF, JEOL, Tokyo, Japan). The cross-sectional microstructure of the coatings prepared by the focused ion beam (FIB) method were investigated using a field-emission transmission electron microscope (FE-TEM; Tecnai G2 F30, FEI, Hillsboro, OR, USA) at an accelerating voltage of 200 kV. Hall measurements were conducted for the electrical resistivity, carrier density, and mobility at room temperature in a Lake Shore system.

### 2.3. Shielding Effects of UV/NIR Coatings on Human Skin Cells (Human Dermal Fibroblasts)

#### 2.3.1. Cell Culture

Human dermal fibroblasts were used to evaluate the UV/IR-induced skin damage. Human dermal fibroblasts (CCD-966SK cell line) obtained from the Bioresources Collection and Research Center (Hsinchu, Taiwan) were cultured in a minimum essential medium (Eagle) supplemented with 10% fetal calf serum, 0.1 mM non-essential amino acids, 1.5 g/L sodium bicarbonate, penicillin (100 units/mL)/streptomycin (100 µg/mL), and 1 mM sodium pyruvate, and incubated at 37 °C in an atmosphere of 95% air and 5% CO_2_.

#### 2.3.2. UV/IR Irradiation

The cells were washed with phosphate buffered saline (PBS) and then irradiated with the UVB light (10 mJ/cm^2^) using UVP Crosslinker CL-3000 (AnalytikJena, Germany). For IR irradiation, PBS-covered cells were exposed to NIR radiation (140 J/cm^2^) generated by a 175 W infrared lamp (IR175R PAR38, Philips, Amsterdam, The Netherlands). No increase in temperature was detected in PBS under these conditions. Control cells were held under similar conditions without irradiation. Following the treatment, the cells were incubated with the culture medium for desired durations at 37 °C.

#### 2.3.3. Evaluation of the Intracellular Reactive Oxygen Species (ROS) Production

H_2_DCFDA (2,7-dichlorodihydrofluorescein diacetate) was employed to detect the intracellular generation of ROS. The irradiated cells were collected and incubated with 10 µM H_2_DCFDA for 20 min prior to harvesting, and then washed with PBS. ROS generation was analyzed by flow cytometry (Beckman FC500, Beckman Coulter, CA, USA).

#### 2.3.4. Real-Time Polymerase Chain Reaction

Total RNA was isolated from the non-irradiated control cells and UV-and/or IR-irradiated cells using the RNeasy MiniKit (Geneaid Biotech Ltd., Taipei, Taiwan) according to the manufacturer. Reverse transcriptase reaction was performed with 2 µg of total RNA using M-MLV reverse transcriptase (Promega, Madison, WI, USA) according to the manufacture’s recommendation. Quantitative PCR was performed using ABI 7500 Real-Time PCR System (Applied Biosystems Inc., Foster City, CA, USA). PCR was performed using the GoTag qPCR Master Mix (Promega). The following thermocycling conditions were used: incubation at 95 °C for 2 min, followed by 40 cycles of amplification at 95 °C for 15 s, and then 60 °C for 60 s. The threshold cycle is defined as the cycle number at which the fluorescence corresponding to the amplified PCR product was detected. The PCR arbitrary units of each gene were defined as the mRNA levels normalized to the glyceraldehyde-3-phosphate dehydrogenase (GAPDH) expression in each sample. The following primer sequences were used: GAPDH (forward primer) 5′-GAAATCCCATCACCATCTTCCAGG-3′, GAPDH (reverse primer) 5′-GAGCCCCAGCCTTCTCCATG-3′, TNF-*α* (forward primer) 5′-AGCCCATGTTGTAGCAAACC-3′, TNF-*α* (reverse primer) 5′-TGAGGTACAGGCCCTCTGAT-3′, IL-6 (forward primer) 5′-CACAGACAGCCACTCACCTC-3′, IL-6 (reverse primer) 5′-TTTTCTGCCAGTGCCTCTTT-3′.

## 3. Results and Discussion

### 3.1. Growth Mechanism and Microstructure of the TiO_2_ Film

**Figure 1:**
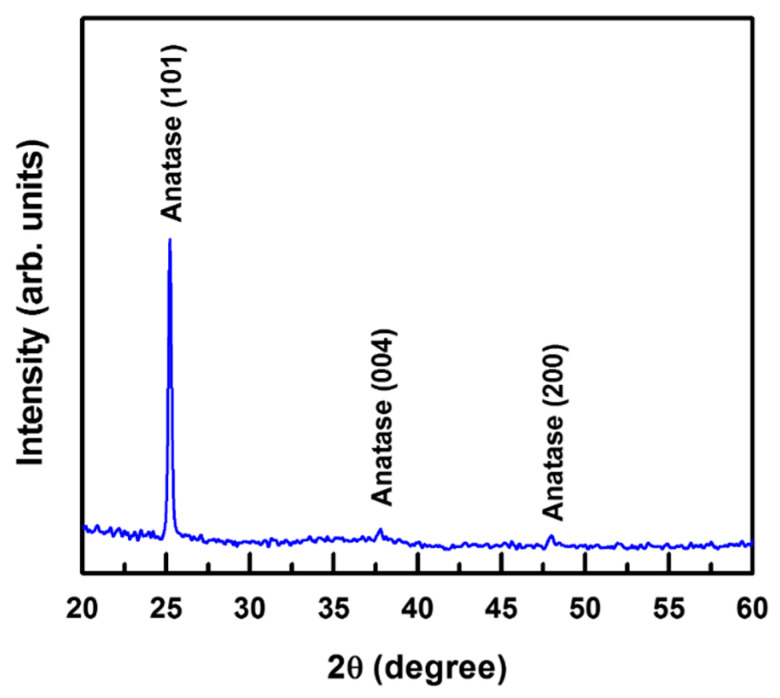
XRD pattern of a 100 nm thick TiO_2_ film deposited on a glass substrate at 100 °C by steered CAPE using a shielded net.

Figure 1 presents the X-ray diffraction (XRD) pattern of a 100 nm thick TiO_2_ film deposited on a glass substrate at 100 °C by CAPE using a shielded net (shielded CAPE) between the arc source and substrate. The diffraction peaks could be attributed to the antase-phase TiO_2_ (JCPDS card No. 21-1272) and the peaks could be indexed to the (101), (004), and (200) planes. Thus, the XRD analysis indicates that single-phase TiO_2_ films can be grown by this method, without any secondary phases. Although the TiO_2_ films were grown at a low temperature of 100 °C, the intensive and sharp diffraction peaks indicated good crystallinity of the coatings. This is attributed to the ion-assisted growth arising from the high-ionization arc plasma [12]. In contrast, the formation of high-crystallinity TiO_2_ films through conventional vapor deposition without ion assistance generally requires a growth temperature of >300 °C [15]. The XRD patterns exhibited a higher intensity of the (101) peak than other peaks, revealing the stronger tendency of the TiO_2_ crystallites to grow with a preferred (101) orientation. According to thermodynamics, a TiO_2_ structure with (101) orientation is more stable because of its lower surface free energy over that of a structure with (100) orientation [28]. Thus, the anatase-phase TiO_2_ films grew preferably along the [101] axis in the direction perpendicular to the glass substrate.

**Figure 2:**
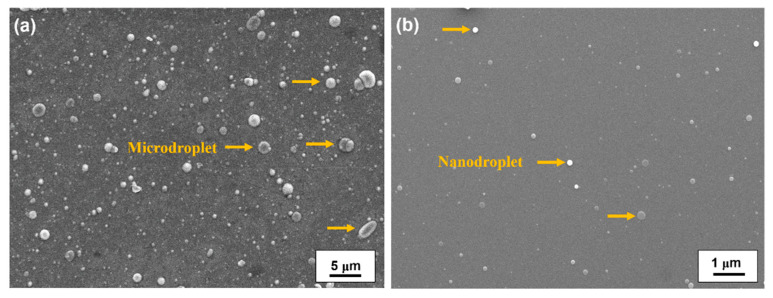
SEM images of TiO_2_ thin films deposited by CAPE: (**a**) without and (**b**) with a shielded net (shielded CAPE) through the reaction of a Ti target with O_2_ at the deposition temperature of 100 °C.

The variations in the surface morphology of the TiO_2_ thin films with the different deposition methods were examined by SEM to investigate the Ti microdroplet size distribution. Figure 2 shows the plane-view SEM images of the TiO_2_ thin films deposited by CAPE (a) without and (b) with a shielded net via the reaction of a Ti target with O_2_ at the temperature of 100 °C. Figure 2a shows some Ti microdroplets incorporated into the TiO_2_ film deposited by CAPE without a shielded net. The size of the microdroplets was in the range of 0.5 to 4 µm, as indicated by the arrows. As the cathode spots are a center of explosive electron emission on the target surface with an extremely high current density (~10^12^ A/m^2^), they create high ion kinetic energies (20–200 eV) and multiple charge states for ion-assisted growth [29]. However, the major disadvantage of this process is the emission of the microdroplets from the target, and their adhesion onto the films during their deposition. Large Ti microdroplets are emitted owing to the interaction between the arc plasma and Ti target at the cathode spots. In general, large microdroplets of sizes ranging up to several micrometers may cause a self-shadowing effect during the thin film growth process, leading to voids or gaps at the film–droplet interfaces [30]. The deposition of large microdroplets on a growing film results in degraded optical properties of the final film, owing to the opacity of the metal microdroplets and self-shadowing effect. These factors restrict the application of the CAPE process in the preparation of optical coatings. To overcome this issue of the incorporation of opaque metal microdroplets into the films, TiO_2_ films are prepared by shielded CAPE via the reaction of a Ti metal target with O_2_. The SEM image of the TiO_2_ film deposited by shielded CAPE (Figure 2b) exhibits only a few Ti nanodroplets incorporated in the film, and the size of the droplets is significantly decreased from several micrometers to 50–150 nm, as marked by the arrows in the image. This result indicates that the liquid droplets of Ti emitted from the target were effectively blocked by the shielded net. However, the average growth rate decreased significantly from 8.5 to 1.2 nm/s when the shielded CAPE method was used.

**Figure 3:**
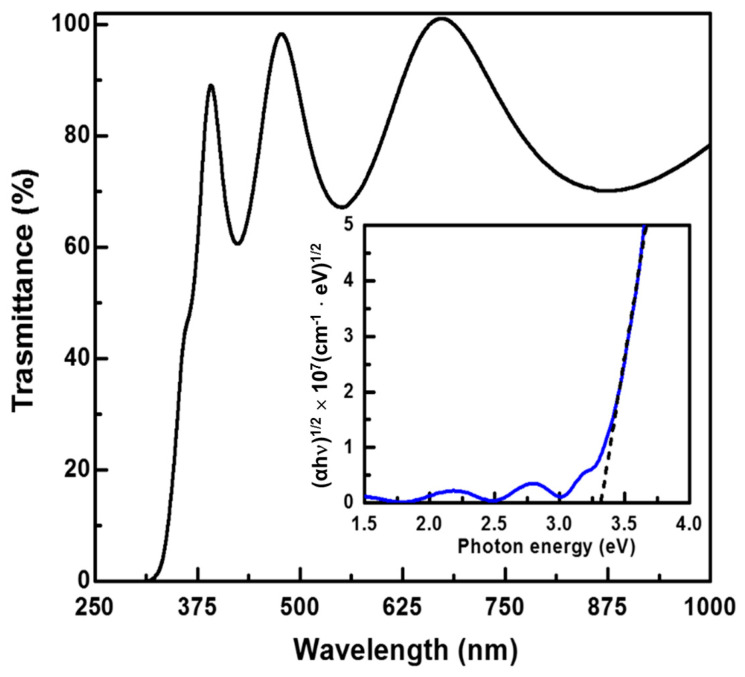
Transmittance spectrum of the TiO_2_ thin film deposited by steered CAPE with a shielded net (shielded CAPE) through the reaction of a Ti target with O_2_ at the deposition temperature of 100 °C. Inset shows the plot of (*α*h*ν*)^1/2^ versus photon energy for the TiO_2_ thin film.

Figure 3 displays the transmittance spectrum of a TiO_2_ thin film deposited by shielded CAPE via the reaction of a Ti target with O_2_ at the growth temperature of 100 °C. The inset shows the optical band gap of the TiO_2_ coating. The series of broad peaks located at 391, 497, and 675 nm is due to light interference owing to the difference in the refractive index between the TiO_2_ thin film and glass substrate; it indicates the uniformity of the TiO_2_ film thickness. The transmittance spectrum indicates that the TiO_2_ films had an average transmittance >83.6% in the visible light range (400–760 nm). The as-deposited TiO_2_ films obtained by shielded CAPE showed good visible light transmittance because the arc plasma provided sufficient energy to Ti and O adatoms to reach their equilibrium positions; thus, the undesired absorption due to structural defects is reduced. In contrast, TiO_2_ films deposited by the DC-sputtering deposition process require a higher thermal treatment temperature of >300 °C to enhance visible light transmittance, because high-temperature thermal annealing is required to eliminate the defects in the film [16].

To investigate the UV-shielding properties of the TiO_2_ films deposited by shielded CAPE, the indirect band gap of the TiO_2_ (E*_g_*_,TiO2_) film was determined from the absorption spectra using the indirect transition model [31]:

(*α*h*ν*)^1/2^ = *A_i_*(h*ν* − E*_g_*_,TiO2_) (1)

where *α* is the absorption coefficient, *A_i_* is a constant, and h*ν* is the photon energy. The inset of Figure 3 shows the plot of (*α*h*ν*)^1/2^ versus the photon energy for the TiO_2_ film. According to Equation (1), the optical bandgap can be determined by extrapolating the linear region of the plot to the abscissa. The bandgap of the TiO_2_ films was thus determined to be ~3.3 eV, which corresponds to the band-to-band transition of anatase-phase TiO_2_. This band-to-band transition (absorption edge at ~375 nm) facilitates a strong absorption in the UV range, leading to high UVB- and UVA-shielding properties.

### 3.2. IRB-Shielding ITO Layers

ITO films have high visible light transparency and good IRB absorption properties owing to the plasmon excitation of the free electrons [27]. ITO films with a high free carrier concentration are prepared carefully to obtain a higher IRB-shielding rate. RF magnetron sputtering combined with a negatively biased substrate can facilitate intense ion bombardment during the film deposition. A suitable bias voltage can provide sufficient kinetic energy to enable the adatoms to reach the equilibrium position, resulting in a highly crystalline film. Therefore, ITO films were deposited on a glass substrate with an RF bias voltage of −40 V at 150 °C.

**Figure 4:**
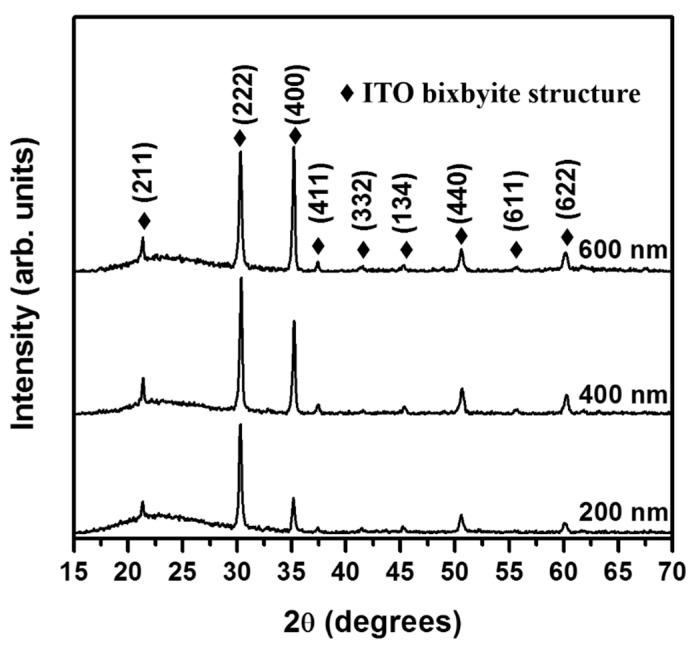
XRD patterns of the ITO thin films, with different thicknesses in the range of 200 nm to 600 nm, deposited by substrate-biased RF magnetron sputtering. The ratio of the (400) to (222) diffraction peak intensity (*I*_400_/*I*_222_) was calculated to be 0.323, 0.678, and 1.03 for the 200, 400, and 600 nm thick ITO films, respectively.

Figure 4 shows the XRD patterns of the ITO films with various thicknesses in the range of 200 to 600 nm. All the recorded diffraction peaks could be indexed to the bixbyite structure of ITO (JCPDS card No. 89-4598). Thus, the XRD analysis indicated that the films were formed in the pure ITO phase, without any secondary phases. Despite the relatively low deposition temperature, the sharp diffraction peaks indicate a good crystallinity of the ITO coatings. This is attributed to enhanced ion bombardment resulting from the effect of the substrate bias. In general, the temperature required for the crystallization of amorphous ITO films is ~300 °C [32]. The ion bombardment provided the adatoms with sufficient energy to move on the growing film surface. Thus, high-crystallinity ITO films could be deposited at a relatively low growth temperature of 150 °C. Furthermore, the intensity ratio of the (400) and (222) diffraction peaks (*I*_400_/*I*_222_) in the XRD patterns increased from 0.32 to 1.03 with an increase in the ITO film thickness from 200 to 600 nm. The *I*_400_/*I*_222_ ratio of the 200 nm thick ITO film was determined to be 0.32, comparable to that of a randomly oriented polycrystalline ITO powder (*I*_400_/*I*_222_ = 0.30) [27]. The orientation of the initially formed ITO layer was influenced by the amorphous structure of glass substrate; therefore, a random orientation was observed when the film thickness was 200 nm. The *I*_400_/*I*_222_ ratio increases significantly from 0.68 to 1.03 as the thickness was increased from 400 to 600 nm, indicating that the (100) preferred orientation evolved with increasing film thickness.

**Figure 5:**
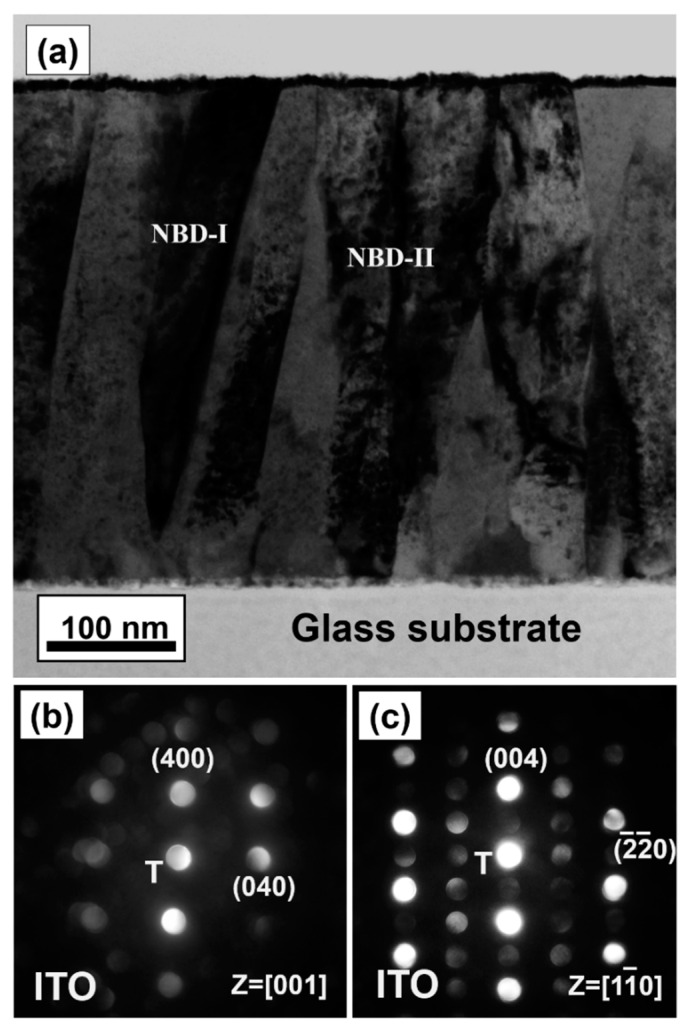
(**a**) A cross-sectional bright-field image transmission electron microscopy (TEM) image of the ITO film deposited on the glass substrate: (**b**,**c**) show the corresponding nano-beam diffraction patterns of regions marked as NBD-I and NBD-II in (**a**), respectively.

To further investigate the microstructure of the ITO films deposited by RF sputtering combined with a negatively biased (−40 V) substrate, a representative cross-sectional sample of a 400 nm thick film was analyzed by TEM (Figure 5). The cross-sectional bright-field (BF) TEM images revealed that the dense columnar structure of ITO film, as shown in Figure 5a. In addition, the initial growth region near the amorphous glass substrate contained small columnar grains (~40 nm) and the columnar grains gradually coalesced into larger columnar grains (~90 nm) with an increase in the film ITO thickness to 400 nm. Figure 5b,c show the nano-beam diffraction (NBD) patterns for the regions marked as NBD-I and NBD-II, respectively, in Figure 5a. The NBD patterns in Figure 5b,c were recorded along the [001] and [$1\bar{1}0$] zone axes, respectively. The NBD could be indexed to the bixbyite-phase ITO with a lattice parameter of 1.016 nm [33]. According to the NBD analysis, the columnar ITO grains grow along the [100] direction, perpendicular to the plane of the substrate. Thus, the BF image and NBD patterns indicates that a (100)-textured microstructure had formed in the ITO films at thicknesses greater than 400 nm, consistent with the XRD results. According to Choi et al., ITO films that contain more oxygen vacancies provide sites for ions to migrate under the low oxygen sputtering growth condition [34]. In addition, the oxygen vacancies are preferentially incorporated into (100) planes than into (111) planes, because the former can accommodate more oxygen vacancies than the latter [33,34]. A large number of oxygen vacancy sites could assist ion diffusion owing to the open lattice structure of these sites. From the perspective of kinetics, only grains with the highest growth-rate lattice plane eventually survive [35]; therefore, the preferred (100) orientation was obtained at a greater film thickness.

**Figure 6:**
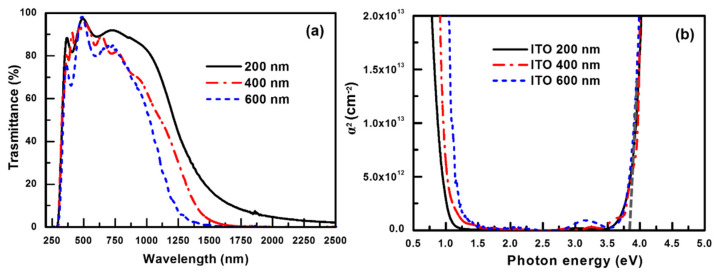
(**a**) Transmittance spectra and (**b**) absorption spectra of ITO films of different thicknesses in the range of 200 to 600 nm.

Figure 6a shows the transmittance spectra of the ITO coatings grown to different thicknesses (200 to 600 nm). The optical properties, including the average transmittance from UVB to UVA (*T_UVB_*_~_*_UVA_*), average visible light transmittance (*T_VIS_*), average IRA transmittance (*T_IRA_*), and average IRB transmittance (*T_IRB_*) were calculated from the transmittance spectra. The electrical and optical properties, including the carrier density, Hall mobility, and resistivity, and the *T_UVB_*_~_*_UVA_*, *T_VIS_*, *T_IRA_*, and *T_IRB_* values of the ITO films with different thicknesses (200–600 nm) are summarized in Table 1. As the film thickness increased from 200 to 600 nm, the Hall mobility increased from 25.9 to 31.8 cm^2^/V s. The carrier density also increased with the film thickness from 9.1 × 10^20^ to 1.12 × 10^21^ cm^−3^. Thus, the best conductivity was obtained at the thickness of 600 nm. The changes in the electrical properties can be rationalized based on the microstructure, crystalline defects and effective donor density. The electrons encountered fewer scattering events during their transport, leading to higher mobility. The mobility increased continuously with an increase in the film thickness from 200 to 600 nm, owing to the decrease in the number of scattering centers due to the reduction in the crystalline defects with increasing crystallite size. The crystallite size and crystallinity of the ITO films increase with an increase in the film thickness from 200 to 600 nm, resulting in the increase in mobility with increasing film thickness.

The high carrier concentration of the high-crystallinity ITO film indicated that the In^3+^ ions were replaced by a Sn^4+^ donors, resulting in the donation of free electrons. Further, the incorporated oxygen vacancies can behave as double donors and the trapped electrons are easily thermally activated to the conduction band [33]. In contrast, grain boundaries and lattice distortion lead to large recombination centers owing to localized states related to the nearby dangling bonds, which could act as undesirable electron-trapping state [36,37]. The carrier concentration increases from 9.1 × 10^20^ to 1.1 × 10^21^ cm^−3^ with increasing thickness from 200 nm to 600 nm. The film with 600 nm thickness has the highest election density, which originates from the increase in the effective dopant concentration due to the improved crystallinity and the decrease in the number of trapping sites arising from crystalline defects. In addition, the ITO films with the preferred (100) orientation at the thickness of >400 nm can incorporate a higher number of oxygen vacancies, leading to higher carrier density.

Figure 6b shows the square of the absorption coefficient versus the photon energy for ITO films of different film thicknesses. The bandgaps of the ITO films can be obtained based on the Moss method [38]. The optical bandgap of the ITO films (E*_g_*_,ITO_) can be determined from the absorption coefficient of the films using the relationship for parabolic direct bands [27]:

*α* = A(h*ν* − E*_g_*,_ITO_)^1/2^ (2)

where *α* is absorption coefficient, A is a constant, and h*ν* is the photon energy. According to Equation (2), the interpolation of the linear part of the *α*^2^ versus h*ν* plot onto the h axis yields the band gap of the ITO films [27]. The E_*g*,ITO_ value determined by this method is ~3.75 eV (absorption edge is located at ~330 nm); thus, ITO films cannot completely block the UVA radiation (wavelength: 320–400 nm). Therefore, the *T_UVB_*_~_*_UVA_* value decreased only marginally from 52.9 to 48.1% owing to the band-to-band absorption of ITO. Moreover, ITO films have a high transmittance of >85% in the visible light range; however, the *T_VIS_* value decreased slightly from 90.9 to 85.2% with increasing film thickness, as shown in Table 2. The visible light transmittance decreased with increasing ITO thickness owing to the broad absorption range of the ITO film, which may be attributed to the s^2^ → sp bonding transition of Sn^2+^ [33]. It has been suggested that the formation mechanism of Sn^2+^ ions involves the charge neutralization; therefore, Sn^2+^ ion were formed to the compensation of the local positive charge at Sn^4+^ in ITO films [33].

Further, the transmittance edge in the NIR region decreased from 1500 to 1250 nm with increasing thickness of the ITO film. The TIRA value decreased rapidly from 66.7 to 38.2%, and the IRB radiation was effectively blocked (i.e., *T_IRB_* < 1%) at the ITO thickness of >400 nm, as shown in Table 1. The transmittance in the IRB region decreased rapidly, because the plasmon resonance absorption was enhanced by the increased free electron density of the TCO film [24,25]. The plasmon resonance frequency (*ω_p_*) is given by *ω_p_* = (4*π*Ne^2^/*ε_o_m*^∗^)^1/2^, where *N* is the density of free electrons, *e* is the elementary charge, *ε_o_* is the permittivity of free space, and *m*^∗^ is the electron effective mass [24,25]. According to the Drude model, the absorption spectrum of plasmons can be described as follows [39]:

*α* = 2*ω*/*c*(*ω_p_*^2^/*ω*^2^ − 1)^1/2^ (3)

where *α* is the absorption coefficient and *ω* is frequency. The absorption coefficient decreases with increasing frequency and it becomes zero for *ω* > *ω_p_*. The carrier concentration of the ITO film increased form 9.1 × 10^20^ to 1.12 × 10^21^ cm^−3^ with an increase in the film thickness from 200 to 600 nm; therefore, the *ω_p_* value shift to high energy when the increase in ITO thickness, as shown in Figure 6b. Despite the good IRB-shielding properties, the cost of indium becomes too prohibitive due to the scarcity of indium. To address the indium-scarcity issues, significant effort has been devoted for development of ITO-alternative materials with high infrared shielding properties, such as gallium-doped ZnO films [25], conductive metal oxides (CMO) plasmonic nanostructures [40], CMO nanocrystals [41], and degenerately doped semiconductor nanocrystals [42].

### 3.3. TiO_2_/SiO_2_ IRA-Shielding Multilayers

The IRB radiation is almost completely absorbed by an ITO film at the thickness of >400 nm. However, as the IR radiation absorption edge of the ITO film is limited to ~1300 nm, the IRA light is not completely shielded by a single ITO layer because of its limited free carrier density. Therefore, to develop a coating with optimal IRA-shielding abilities, a series of TiO_2_/SiO_2_ multilayer films with different stacking structure was designed. NIR-reflecting multilayer coatings composed of the high-refractive-index TiO_2_ and low-refractive-index SiO_2_ films were deposited on glass substrates using the shielded CAPE and RF sputtering deposition processes, respectively. As the IRA waves undergo constructive interference, the thickness of a TiO_2_/SiO_2_ alternating multilayer is set to be equal to the quarter of the IRA wavelength [43]. The thickness (*t*) values of the individual TiO_2_ and SiO_2_ films determined using the relationship *t* = *λ_B_*/4*n* [43], where n is the refractive index (TiO_2_: 2.25, SiO_2_:1.45) and *λ_B_* is the Bragg wavelength. TiO_2_(SiO_2_/TiO_2_)*_x_* stacked multilayers with TiO_2_/SiO_2_ pairs (*x*) equal to 1–3 are designed to fulfill a Bragg wavelength (*λ_B_*) of ~950 nm. According to a quarter of the Bragg wavelength relationship (*t* = *λ_B_*/4*n*), the thickness of each oxide layer was 163.8 and 105.6 nm for SiO_2_ and TiO_2_, respectively.

**Figure 7:**
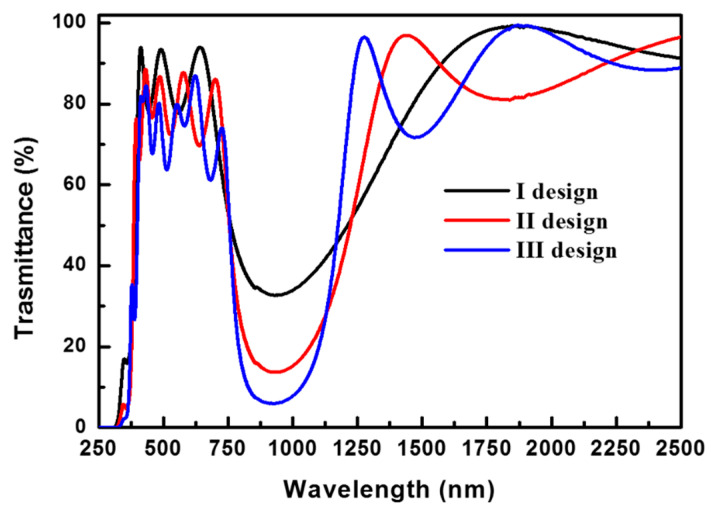
Transmittance spectra corresponding to various optical designs, viz., (I) TiO_2_ (SiO_2_/TiO_2_), (II) TiO_2_(SiO_2_/TiO_2_)_2_, and (III) TiO_2_(SiO_2_/TiO_2_)_3_ with a Bragg wavelength of 950 nm.

Figure 7 shows the transmittance spectra of the coatings with various structures that fulfill a Bragg wavelength of 950 nm, viz., (I) TiO_2_(SiO_2_/TiO_2_), (II) TiO_2_(SiO_2_/TiO_2_)_2_, and (III) TiO_2_(SiO_2_/TiO_2_)_3_. Table 2 lists the average *T_VIS_*, *T_IRA_*, and *T_IRB_* values of the SiO_2_/TiO_2_ multilayers, viz., structures (I), (II), and (III). The *T_VIS_* (i.e., the transmittance in the visible light region) decreases from 81.6 to 72.7% with an increase in the number of pairs (x) in the order of (I) > (II) > (III). The *T_IRA_* significantly decreases from 45.1 to 38.2% with an increase in the number of TiO_2_/SiO_2_ pairs from 1 to 3, which attributes to the increase in the number of reflections, and the constructive interference of the multiply reflected waves originating from the structure with an increased number of TiO_2_/SiO_2_ pairs [43]. The TiO_2_/SiO_2_ multilayer shielded the IRA light; however, the transmittance spectra of the optical structures denoted as (I), (II), and (III) indicated high transmittances (>88.8%) in the IRB region (as shown in Table 2). The band width of a TiO_2_/SiO_2_ multilayer depends on the difference in the refractive index of its constituent materials; a greater difference results in a greater IRA-shielding band width, while an increase in the number of pairs may narrow the band width. The TiO_2_(SiO_2_/TiO_2_)_3_ multilayer (design III) exhibited the best IRA-shielding abilities; however, its band width was only ~420 nm in the IRA wavelength range of 760–1200 nm, as indicated in Figure 7. Therefore, the NIR radiation was not completely shielded by the single-Bragg wavelength TiO_2_(SiO_2_/TiO_2_)*_x_* multilayer owing to the constrained difference between the refractive indices of the constituent layers.

**Figure 8:**
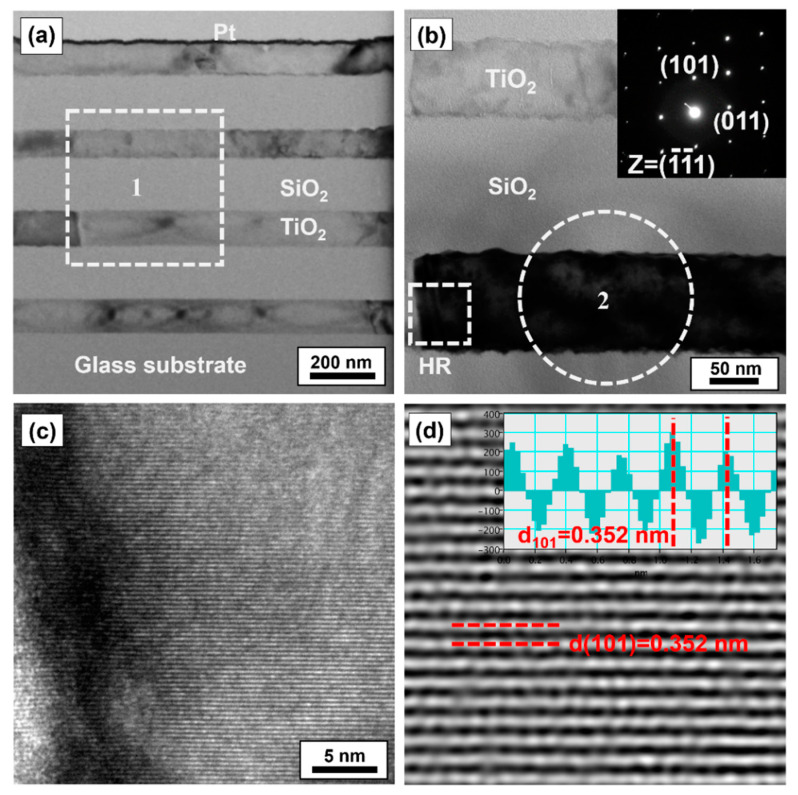
(**a**) A cross-sectional bright-field transmission electron microscopy (TEM) image of an IRA-shielding TiO_2_(SiO_2_/TiO_2_)_3_ multilayer film with a Bragg wavelength of 950 nm deposited on a glass substrate. (**b**) A High-magnification TEM image of region 1 in (**a**). Inset shows a diffraction pattern of the anatase-phase TiO_2_ grains from region 2 in (**b**). (**c**) High-resolution TEM image of the HR region in (**b**). (**d**) A two-dimensional inverse fast Fourier transform image of the HR-TEM image.

To study the effects of the microstructure on the optical properties of the TiO_2_/SiO_2_ IRA-shielding multilayer films, representative cross-sectional specimens prepared by the FIB method for a TiO_2_/SiO_2_ multilayer with design (III) was analyzed by TEM. Figure 8a shows a BF TEM image of a SiO_2_/TiO_2_ multilayer with design (III) deposited on a glass substrate. The BF image clearly shows that the SiO_2_ and TiO_2_ layer are dense and uniform. The interfaces of the multilayers are sharp, indicating that the inter-diffusion of atoms across the SiO_2_/TiO_2_ interfaces is not significant owing to the low growth temperature. In order to identify the phase and investigate the orientation of the TiO_2_ grains, Figure 8b shows a magnified BF TEM image for the square region marked as 1 in Figure 8a, and the corresponding selected-area diffraction pattern (SADP) of the TiO_2_ film is shown in the inset of Figure 8b, as indicated for region 2 in Figure 8b. The SADP was recorded along the [$\bar{1}\bar{1}1$] zone axis and the SADP could be indexed to the anatase-phase TiO_2_ with the lattice parameters of a = 0.3785 nm and c = 0.9513 nm [8]. The SADP of the film deposited on amorphous SiO_2_ showed (101) preferred orientation, indicating that the TiO_2_ crystallites grow along the [101] axis. The magnified BF TEM image and SADP unequivocally demonstrate that a highly crystalline TiO_2_ film was obtained by the shielded CAPE process at a low growth temperature. Therefore, the TiO_2_ films with high visible-light transparency and high refractive index (2.25) were obtained.

In general, the deposition temperature required to form highly crystalline TiO_2_ films is greater than 400 °C [15]. This is attributed to the ion-assisted growth resulting from the high-density plasma of CAPE. Previously, the kinetics of the crystallization of amorphous TiO_2_ to the anatase phase has been studied by differential scanning calorimetry, and the activation energy of crystallization has been determined to be 202.4 kJ/mol (~2.1 eV/atom) [44]. In the arc plasma, each ion has kinetic and potential energies, which contribute to the energy delivered to adatoms on the growing film, a process referred to as atomic scale heating (ASH) [45]. The ionization energy of multiple charged ions contributes predominantly to the potential energy in the CAPE process, and the ionization energy is in the range of several electron volts to hundreds of electron volts depending on the charge state of the ion [45]; note that this ionization energy is much higher than that of the thermal evaporation or magnetron sputtering techniques [12]. The activation energies for the surface diffusion of the adatoms of Ti and O on the TiO_2_ surface are typically ~0.5 eV [46] and ~2 eV [47], respectively. These activation energies for the surface diffusion of the adatoms on growing films are smaller than those provided by the ASH in the cathodic arc plasma; this results in enhanced surface mobility during the deposition process.

Figure 8c shows a high-resolution (HR) TEM image of the square region marked as HR in Figure 8c. Figure 8d shows a two-dimensional inverse fast Fourier transform (2D IFFT) image corresponding to Figure 8c. The 2D IFFT image indicates the planes of the anatase TiO_2_ (101) planes (viewed edge on) with an interplanar spacing of 0.35 nm, suggesting that ideal (line- and planar-defect-free) TiO_2_ crystallites were grown in the [101] direction. Moreover, the (101) lattice plane grew perpendicular to the film growth direction, leading to a (101)-textured microstructure. As the (101) plane of TiO_2_ is thermodynamically stable because of its lower surface free energy than those of other planes [28], the adatoms on the (101) crystalline plane diffuse faster, resulting in the growth of grains with (101) orientation. These results are consistent with the XRD results of the TiO_2_ films.

### 3.4. UV/NIR-Shielding Double-Sided Coatings

**Figure 9:**
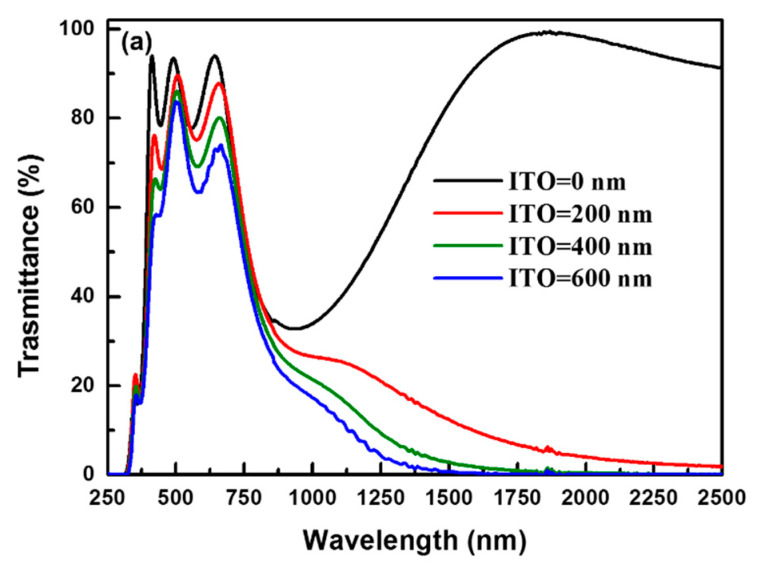

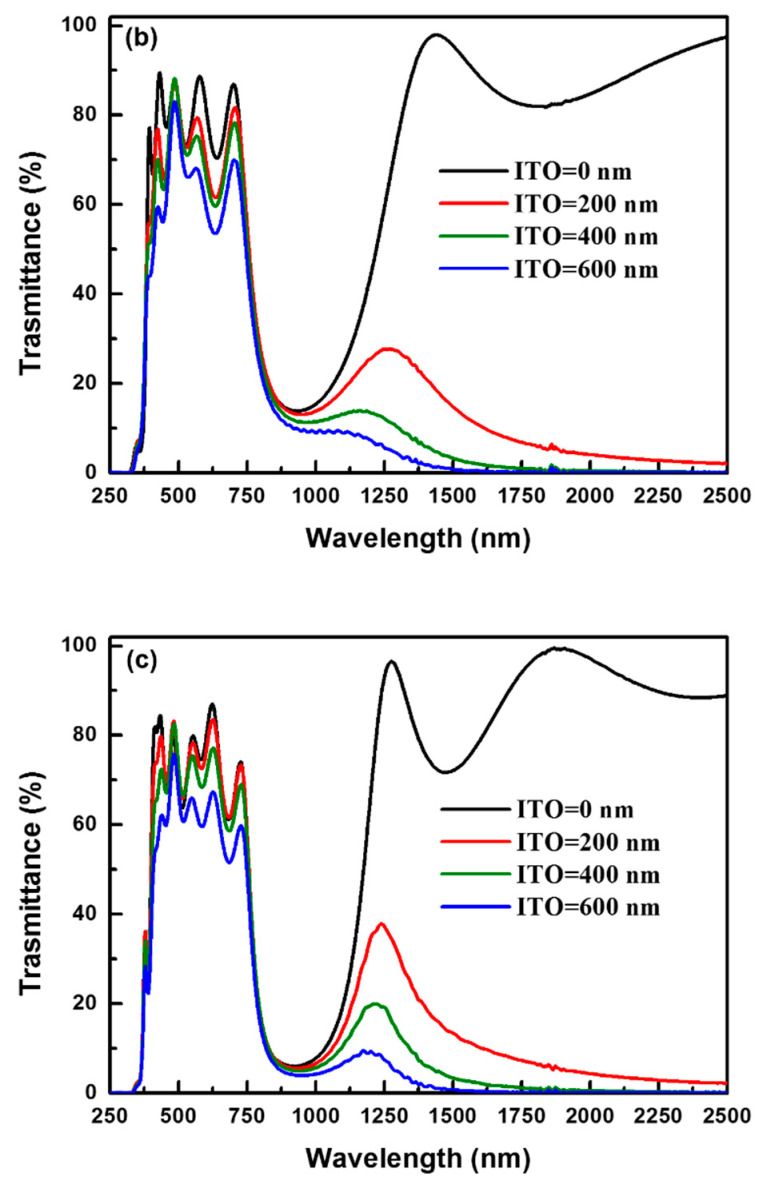
Transmittance spectra of various optical designs, namely (**a**) TiO_2_(SiO_2_/TiO_2_), (**b**) TiO_2_(SiO_2_/TiO_2_)_2_, and (**c**) TiO_2_(SiO_2_/TiO_2_)_3_ with various ITO film thicknesses (0~600 nm).

To obtain a better UV-shielding rate and wide-range NIR-shielding ability of the coatings; thus, UV/NIR-shielding double-sided coatings were fabricated. These double-sided UV/NIR coatings consist of quarter-wave stacks of SiO_2_/TiO_2_ multilayers and ITO films with different thicknesses (200–600 nm) on different side of glass. Figure 9 shows the transmittance spectra of the different optical structures, viz., (I) TiO_2_(SiO_2_/TiO_2_), (II) TiO_2_(SiO_2_/TiO_2_)_2_, and (III) TiO_2_(SiO_2_/TiO_2_)_3_ with different ITO film thicknesses (0–600 nm). Table 3 summarizes the key parameters used in the evaluation of the performance and characteristics of the different designs with various ITO film thicknesses in the range of 0 to 600 nm. That is, the average UV transmittance in the range of UVB to UVA (*T_UVB_*_~_*_UVA_*), average visible light transmittance (*T_VIS_*), average IRA transmittance (*T_IRA_*), average IRB transmittance (*T_IRB_*), average NIR (wavelength: 760–2500 nm) transmittance (*T_NIR_*), UVB to UVA-shielding rate (*S_UVB_*_~_*_UVA_* = 100% − *T_UVB_*_~_*_UVA_*), NIR-shielding rate (*S_NIR_* = 100% − *T_NIR_*), figure of merit (*S_UVB_*_~_*_UVA_* × *T_VIS_* × *S_NIR_*), and transparent thermal insulation index (*K*) of the SiO_2_/TiO_2_ multilayers of designs (I), (II), and (III) are compared. In the evaluation of a coating, the UV-shielding properties in the UVB to UVA range, visible light transmittance, and NIR-shielding ability should be taken into consideration; therefore, the product of *S_UVB_*~*UVA*, *T_VIS_*, and *S_NIR_* (*S_UVB_*~*UVA* × *T_VIS_* × *S_NIR_*) is defined as the figure of merit (FOM). A larger FOM value indicates both a better transparency in visible light and a better UV/NIR-shielding performance. The *T_UVB_*_~_*_UVA_* value not only decreased with increasing in the number of SiO_2_/TiO_2_ pairs, but also with an increase in the ITO film thickness. The enhancement of the UV-shielding properties is due to the band-to-band absorptions of the anatase TiO_2_ and ITO films. Therefore, the TiO_2_(SiO_2_/TiO_2_)_3_ multilayer coating with different ITO film thicknesses (0–600 nm) exhibited *S_UVB_*_~_*_UVA_* values of >90%.

The *T_VIS_* values of all the optical designs decreased with the increase in the ITO film thickness from 0 to 600 nm. This decrease in the visible light absorption may be attributed to the s^2^ → sp bonding transition of Sn^2+^ [33]. The *S_NIR_* of the UV/NIR-shielding films increased with increasing ITO film thickness. The IRB radiation was almost completely shielded (i.e., *T_IRB_* ≤ 1%) at the ITO film thickness of >400 nm. This is because ITO has high-density free carriers, resulting in plasmon resonance absorption in the IR range. Therefore, the ITO thickness should be restricted to achieve a balance between the transparency in visible light and NIR- and UV-shielding rate. All the structures with an ITO thickness of 400 nm showed a high NIR-shielding rate (*S_NIR_* > 92%) and a high *T_VIS_* value (>68%). The three types of the optical designs combined with the 400 nm ITO film yielded a higher FOM (*S_UVB_*_~_*_UVA_* × *T_VIS_* × *S_NIR_*) of >0.58, which indicates their better transparency and UV/NIR-shielding properties over other UV/NIR-shielding coatings with ITO film thicknesses of 200 and 600 nm. Among the metal-free double-side shielding coatings, 400 nm thick ITO films combined with TiO_2_(SiO_2_/TiO_2_)_3_ multilayers with the optical structure of (III) yielded the highest figure of merit (0.60) the corresponding average visible light transmittance is 68.1%, near-infrared shielding rate is 94.8%, and UVB to UVA-range shielding rate is 92.7%.

To compare the transparent thermal insulation performance of the transparent UV/NIR-shielding films with recent studies of transparent IR-sheading materials, the transparent thermal insulation index was used. The transparent thermal insulation index (*K*) is defined as the ratio of the average visible light transmittance (*T_VIS_*) to the average NIR transmittance (*T_NIR_*) [48,49], expressed as follows:

*K = T_VIS_/T_NIR_* (4)

A higher *K* value implies better performance and the *K* values of the different coatings in this work are listed in Table 3. In general, the typical *K* values for Cs_x_WO_3_-based films with excellent transparent thermal insulation performance are in the range of 7–9 [48,49]. Table 3 summarizes the *K* values of the UV/NIR-shielding coatings prepared in this study. The *K* values of our UV/NIR-shielding coatings not only increased with the increase in the ITO film thickness from 0 to 600 nm, but also with an increase in the number of SiO_2_/TiO_2_ pairs. The high-performance transparent thermal insulation coatings of different optical structures with a *K* value of >9.43 could be obtained using an ITO film thickness of >400 nm. Among the prepared oxide-based shielding coatings, 600 nm thick ITO films combined with the TiO_2_(SiO_2_/TiO_2_)_3_ multilayers (optical design (III)) yielded the highest *K* value (20.98), which is better than those of the Pt-doped Cs_x_WO_3_ nanorods (*K* = 8.79) [48] and two-dimensional nano-Cs*_x_*WO_3_ films (*K* = 7.1) [49]. According to the *K* value analysis, it can be concluded that the UV/NIR-shielding coatings developed in this study have excellent thermal insulation performance, and can be prepared at a low deposition temperature of <150 °C. The excellent transparent thermal insulation performance of the UV/NIR-shielding coatings along with high visible transparency and NIR-shielding performance renders them suitable to be applied as energy-saving window coatings in buildings and automobiles.

### 3.5. Effects of UVB/IR Radiations on Human Skin Cells (Human Dermal Fibroblasts)

**Figure 10:**
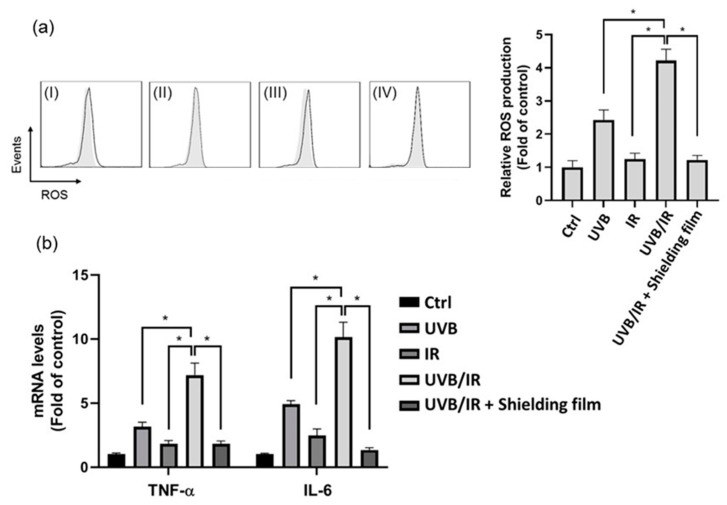
Effect of UVB/IR radiation on human dermal fibroblasts. (**a**) Effect of the UVB/IR radiation with or without the use of a shielding film on the ROS generation of CCD-966SK. The cells were irradiated with 10 mJ/cm^2^ UVB light, 140 J/cm^2^ IR light, or UVB/IR without/with the use of a shielding film. (Left panel) A flow cytometry histograms of DCFDA staining in controls (gray filled histograms) and treatments (black line histograms). (Right panel) Relative fluorescence intensities from flow cytometry (n = 3, mean ± SD, * *p* < 0.05). (**I**) With UVB irradiation, (**II**) with IR irradiation, (**III**) with UVB/IR irradiation, (**IV**) with UVB/IR irradiation through the shielding film. (**b**) Effect of the UV/IR irradiation with or without the use of shielding film on the TNF-*α* and IL-6 mRNA levels. The mRNA levels were analyzed by real-time PCR (n = 3, mean ± SD, * *p* < 0.05).

Photoaging is the most crucial factor on causing skin aging damage. The light-induced ROS generation elicits inflammatory cytokines releasing, DNA modification and oxidative damage. Previous study has revealed that UVB-induced skin is damaged by the excess of ROS generation in human dermal fibroblasts [50]. NIR radiation induced cellular senescence in human keratinocyte, which resulted in premature skin aging [51]. Moreover, the IR radiation reduces the amount of the endogenous antioxidant enzyme catalase and superoxide dismutase, leading to the deterioration of the antioxidant defense capability against oxidative stress [52]. The effect of the UVB or IR or UVB/IR radiation on the ROS generation was therefore examined. Human dermal fibroblast cell line CCD-966SK cells were exposed to UVB or IR or UVB/IR, and intracellular ROS levels were determined DCF fluorescence using flow cytometry. As shown in Figure 10a, a 2.48-fold increase in the ROS generation was noted when the human dermal fibroblasts were exposed to UVB radiation. No significant change in the ROS generation was observed with IR-irradiated cells. The mean intensity following the H_2_DCFDA staining was significantly higher in the UVB/IR-irradiated cells than in the UVB-irradiated cells, consistent with increased ROS generation in the UVB/IR-irradiated cells (4.22-fold increase), suggesting that the increased production of ROS in the UVB/IR-exposed human fibroblasts is related to the IR irradiation-induced decrease in the antioxidant defense capability of the skin. Previous studies have revealed that increased oxidative stress in the skin leads to inflammatory responses, which involve the production of various inflammatory cytokines such as TNF-*α* and IL-6 [53]. Real-time PCR analyses revealed that the amounts of TNF-*α* mRNA and IL-6 mRNA expressed in the UVB/IR-irradiated cells are higher than that in the UVB- or IR-irradiated cells (Figure 10b), suggesting that the oxidative stress causes the increase in the expression of the inflammatory cytokines, TNF-*α* and IL-6.

The metal-free UV/NIR-shielding coatings were also evaluated for their ability to protect human skin cells from UVB/IR through ROS generation and inflammatory cytokine (TNF-*α* and IL-6) mRNA up-regulation in human dermal fibroblasts. We selected the structure with the highest figure of merit and K value, TiO_2_(SiO_2_/TiO_2_)_3_ multilayers combined with the 600 nm ITO film, to study the influence of the UVB/IR-shielding coating on the dermal fibroblasts. As shown in Figure 10a, the ROS generation of the fibroblasts protected by the UV/NIR-shielding film was lower than that of the unshielded UVB/IR-exposed fibroblasts. This result suggested that the UV/NIR-shielding film significantly decreased the UV/IR transmittance, thereby decreasing the deleterious effects of the UVB/IR radiation through ROS generation. Moreover, these cells displayed TNF-*α* and IL-6 mRNA up-regulation after a 24 h exposure to UV/IR irradiation. However, when protected with the UV and NIR-shielding film, they exhibited reduced expression of the UVB/IR-induced inflammatory cytokines (Figure 10b). These data demonstrate that the metal-free UV/NIR-shielding coatings effectively suppressed the UVB/IR-induced ROS generation and inflammatory responses in a human dermal fibroblast cell line. Thus, this work provides practical applications of metal-free high-performance UV/NIR-shielding coatings for human skin protection.

## 4. Conclusions

We employed a novel method to fabricate a metal-free UV/NIR-shielding coating with high IR- and UV-shielding performance and high transmittance in visible light for energy-saving applications and human skin protection. These double-sided coatings consist of quarter-wave stacks of SiO_2_/TiO_2_ multilayers and ITO films on different side of glass, which were responsible for blocking the IRA and IRB radiations, respectively. The total thicknesses of UV/near infrared-shielding films are in the range from 375 nm to 1513.8 nm. The high-performance UV-shielding properties are due to the band-to-band absorptions of the anatase TiO_2_ and ITO films. TEM analysis revealed that the SiO_2_/TiO_2_ multilayers had a uniform and dense structure. A highly crystalline TiO_2_ film with a (101) preferred orientation was obtained by the shielded CAPE process at a low growth temperature of 100 °C. The ITO films were highly crystalline and their free electron concentration increased from 9.2 × 10^20^ to 1.12 × 10^21^ cm^−3^ with increasing thickness, leading to strong absorption of IRB due to the plasmon resonance absorption. Among the metal-free shielding coatings studied, coatings with a 400 nm thick ITO film combined with TiO_2_(SiO_2_/TiO_2_)_3_ multilayers (optical design (III)) yielded the highest figure of merit (0.60); the corresponding average transmittance in visible light is 68.1%, and the NIR shielding rate is 96% and UVB to UVA range shielding rate is 92.7%. According to the transparent thermal insulation index evaluation, 600 nm thick ITO films combined with the TiO_2_(SiO_2_/TiO_2_)_3_ multilayers (optical design (III)) has the highest K value (20.98). The effects of the UV/NIR radiation on human skin cells indicated a significantly reduced ROS generation and inflammatory cytokine expression of the skin cells protected by a UV/NIR-shielding coating as compared to those of the unprotected specimens.

## Figures and Tables

**Table 1:** Carrier density, Hall mobility, resistivity, average transmittance in the UVB to UVA range (*T_UVB_*_~*UVA*_), average visible light transmittance (*T_VIS_*), average IRA light transmittance (*T_IRA_*), and average IRB light transmittance (*T_IRB_*) of ITO films with different thicknesses (200–600 nm).

ITO Thickness (nm)	Carrier Density (×10^20^/Cm^3^)	Hall Mobility (cm^2^/V·Sec)	Resistivity (×10^−4^)	*T_UVB~UVA_* (%)	*T_VIS_* (%)	*T_IRA_* (%)	*T_IRB_* (%)
200	9.1	25.9	2.65	52.9	90.9	66.7	7.3
400	10.5	30.4	1.96	50.9	87.5	50.3	0.9
600	11.2	31.8	1.75	42.1	85.2	38.2	0.2

**Table 2:** Average visible light transmittance (*T_VIS_*), average IRA light transmittance (*T_IRA_*), and average IRB light transmittance (*T_IRB_*) of different optical designs, (I), (II), and (III). The multilayers consist of stacks of (I) TiO_2_(SiO_2_/TiO_2_), (II) TiO_2_(SiO_2_/TiO_2_)_2_, and (III) TiO_2_(SiO_2_/TiO_2_)_3_ layers with a Bragg wavelength of 950 nm.

Optical Design	*T_VIS_* (%)	*T_IRA_* (%)	*T_IRB_* (%)
I	81.6	45.1	93.5
II	77.8	38.5	89.3
III	72.7	38.2	88.8

**Table 3:** Average UV light transmittance in the range of UVB to UVA (*T_UVB_*_~*UVA*_), average visible light transmittance (*T_VIS_*), average IRA light transmittance (*T_IRA_*), average IRB light transmittance (*T_IRB_*), average near IR light (wavelength: 760–2500 nm) transmittance (*T_NIR_*), UVB to UVA shielding rate (*S_UVB_*~*UVA* = 100% − *T_UVB_*~*UVA*), near-IR shielding rate (*S_NIR_* = 100% − *T_NIR_*), figure of merit (*FOM = S_UVB_*_~*UVA*_ × *T_VIS_* × *S_NIR_*) value, and transparent thermal insulation index (*K*) of UV/NIR-shielding double-sided coatings with different optical designs and different thicknesses of the ITO film (200–600 nm).

Optical Design	ITO Thickness (nm)	*T_UVB~UVA_* (%)	*T_VIS_* (%)	*T_IRA_* (%)	*T_IRB_* (%)	*T_NIR_* (%)	*S_NIR_* (%)	*S_UVB~UVA_* (%)	*FOM*	*K*
I	0	16.0	81.6	45.1	93.5	75.7	24.3	84.0	0.17	1.08
	200	13.0	76.8	26.4	5.8	13.4	86.6	87.0	0.58	5.75
	400	12.0	71.2	18.9	0.7	7.6	92.4	88.0	0.58	9.43
	600	10.5	66.0	14.6	0.2	5.5	94.5	89.5	0.56	11.93
II	0	14.1	77.8	38.5	89.3	70.5	29.5	85.9	0.20	1.10
	200	12.6	72.0	21.3	6.4	11.9	88.1	87.4	0.55	6.07
	400	11.9	69.3	13.8	0.8	5.7	94.3	88.1	0.58	12.07
	600	10.4	62.6	9.9	0.2	3.8	96.2	89.6	0.54	16.51
III	0	9.4	72.7	38.2	88.8	70.6	29.4	90.6	0.19	1.03
	200	7.9	71.7	19.0	6.4	11.0	89.0	92.1	0.59	6.52
	400	7.3	68.1	12.2	0.8	5.2	94.8	92.7	0.60	12.97
	600	6.2	59.8	7.4	0.2	2.9	97.1	93.8	0.55	20.98
